# Microstructure and Mechanical Performance of Graphene Nanosheets Reinforced Nickel-Based Superalloy FGH95 Composite

**DOI:** 10.3390/nano10010100

**Published:** 2020-01-03

**Authors:** Yuxi Gao, Jinwen Zou, Xuqing Wang, Xiaofeng Wang, Jie Yang, Huaming Wang

**Affiliations:** 1 School of Materials Science and Engineering, Beihang University, Beijing 100191, China; wogayin@163.com; 2 Science and Technology on Advanced High Temperature Structural Materials Laboratory, AECC Beijing Institute of Aeronautical Materials, Beijing 100095, China; zoujinwen115@163.com (J.Z.); wangxuqing621@163.com (X.W.); wangxiaofeng621@163.com (X.W.); yangjie@163.com (J.Y.)

**Keywords:** nickel based superalloy, graphene nanosheet, mechanical properties, failure behavior

## Abstract

Graphene nanosheet (GNS)-reinforced nickel-based superalloy FGH95 (GNSs/FGH95) matrix composites are prepared via the powder metallurgy approach. Scanning electron microscopy, transmission electron microscope and static tensile tests are used to investigate the microstructure and mechanical properties of GNS-reinforced nickel-based superalloy FGH95. Mechanical properties and failure behavior at room temperature and high temperature are studied. Static tensile tests at room temperature and high temperature confirm that the strength and plasticity of GNS-reinforced FGH95 have been improved, compared to the unreinforced superalloy. The results show that with the increase of temperature, the failure behavior of GNSs/FGH95 composite changes from the interface debonding of the GNSs/matrix to the failure of the FGH95 matrix. This work suggests that GNSs/FGH95 composite has great potential to be a structural material in aero-engine fields.

## 1. Introduction

In recent years, graphene [1,2] with a two-dimensional single-layer structure has been the focus of study, due to its unique mechanical strength [3] and Young’s modulus [4], high electrical conductivity [5] and good thermal conductivity [6]. Compared to single-layer graphene, graphene nanosheets (GNSs) [7,8] consisting of a few layers of graphene are relatively easy to prepare and have similar properties to the single-layer graphene. It has been widely investigated that the addition of GNSs could improve the mechanical properties of polymers [9] and ceramics [6]. More recently, metal matrix composites [10,11] enhanced with GNSs have drawn much attention, for the possibility to combine the excellent properties of graphene with metal to meet with the requirements of high strength [12,13] and good toughness [14,15]. For instance, Rashad et al. [16] successfully fabricated graphene-reinforced magnesium composites and found that graphene embedded in magnesium matrix led to an increase in Young’s modulus (+131%), yield strength (YS) (+49%) and fracture strains (+74%) of the composites. Wang et al. [17] proposed an in situ reaction wetting strategy for graphene-reinforced magnesium composites and found that with the addition of 0.3 vol.% graphene, the YS, tensile strength and hardness of the composite increased by 63%, 17% and 56%, respectively. Yang et al. [18] revealed that the content of graphene determined the ultimate tensile strength (UTS) and bending strength performance of the graphene/Al-20Si composite. With the addition of 1.5 wt.% graphene, the mechanical properties hit the peak value, which increased 130% and 230% compared to the unreinforced matrix, respectively. Khobragade et al. [19] fabricated graphene-reinforced copper matrix composite, which demonstrated that with 10 wt.% graphene content, the hardness and Young’s modulus increased by 118% and 101%, respectively. Mu et al. [20] investigated how the multi-layer graphene-reinforced pure titanium and the compressive YS exhibited more than two times higher the pure one.

Regarded as the most widely applied structural material in aircraft and the aerospace field, nickel-based superalloy has been used in large quantities due to the good tensile and creep properties and high temperature stability for extended periods of exposure. However, with the rapid development of the aerospace industry and the gradual increase in the demand for extreme working environments, the existing nickel-based superalloy cannot meet the requirements of the harsh working condition. Besides, it is very difficult to achieve a significant increase of mechanical performance through a traditional process [21,22,23], such as heat treatment, deformation process and alloy components adjustment. Graphene, as the thinnest material with outstanding physical and mechanical properties, can be regarded as a prospective reinforcing material in various engineering materials. Thus, it is possible to impart additional properties to the nickel matrix by producing a metal matrix composite [24]. Fu et al. [25] fabricated nickel matrix composites reinforced by three-dimensional graphene networks. With 1.0 vol% graphene addition, the YS and UTS of the composites increased by 188.4% and 26.0%, respectively. Ma et al. [26] prepared the reduced graphene oxide-reinforced Inconel 718 through spark plasma sintering, and obtained 19.17% higher ultimate compressive strength than the unreinforced sample. A nickel matrix reinforced with graphene [27,28,29,30] has demonstrated considerable improvement in mechanical behavior compared to the monolithic metal matrix. Graphene could be used as an ideal dispersion strengthening phase [31], to improve the mechanical response of nickel matrix composites. However, due to the large difference in physical and chemical properties between graphene and superalloy [32,33], there are still many barriers for the further development of graphene-reinforced nickel-based superalloy, such as the interface combination between graphene and metal matrix and fracture mechanism at different temperatures. Therefore, continuous efforts are required to incorporate graphene in composite materials to get improved performance properties of nickel based superalloy for practical applications.

To the best of our knowledge, limited work has focused on the high-temperature mechanical properties and fracture mechanisms at different temperatures of a nickel-based superalloy matrix composite reinforced with GNSs until now. In this paper, the nickel-based superalloy FGH95 reinforced with GNSs (GNSs/FGH95) was fabricated via the powder metallurgy technique. This method can improve the performance of metal matrix composites by maintaining the integrity of the graphene structure. The tensile properties and fracture mechanism at room temperature and 650 °C based on different GNSs content-reinforced FGH95 matrix composites were explored. The experimental results revealed that the addition of GNSs can enhance the mechanical properties of nickel-based superalloy and the graphene-reinforced composite has the potential to become a new generation of structural materials.

## 2. Experimental

**Figure 1:**
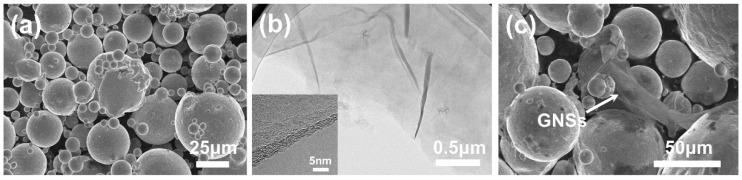
Scanning electron microscope (SEM) image of FGH95 powder (**a**); transmission electron microscope (TEM) images of as-prepared graphene nanosheets (GNSs) (**b**); SEM image of the FGH95 powders with GNSs (with 0.01 wt.% GNSs) after mechanically stirred (**c**). The inset in (**b**) is the TEM image of GNSs layers.

Nickel-based superalloy FGH95 powder was prepared by the gas atomization method [34] in the atmosphere of argon which has a spherical morphology with mean diameter size of ~50 μm, as illustrated in the scanning electron microscope (SEM) in Figure 1a. Graphene oxide was synthesized via Hummer’s method [35], then chemically reduced to GNSs by heating at 450 °C for 18 h. The GNSs prepared by this method has a two-dimensional high aspect-ratio nanosheet type and large lateral size with some wrinkles, as shown in the transmission electron microscopy (TEM) images in Figure 1b. The corresponding thickness of the as-prepared GNSs is about 2.7 nm–3.5nm, it can be estimated that the layer number of the as-prepared GNSs would be 8–10 stacking sheets [36], seen from the inset in Figure 1b.

The preparation process of FGH95 powders mixed with GNSs mainly contains five main steps: mingling, degassing, hot isostatic pressing (HIP), isothermal forging (HIF) and heat treating (HT). Firstly, the GNSs were mixed with FGH95 powder via a wet chemical mechanically stirring method, ethanol can be used as solvent. The mixed powder was mechanically stirred at 80 °C in an oil bath until it turned to be semi-dry condition, then the mixture was completely dried in an oven at 70 °C. Figure 1c shows the morphology of FGH95 powder with GNSs after mixing. The image shows that GNSs have been adhered to the surface of FGH95 powder and maintain their original structure with no obvious GNSs agglomerations in the mixture. The addition amounts of the GNSs in the FGH95 mixed powder were 0.01 wt.% and 0.03 wt.%, respectively. Secondly, the mixed powders were encapsulated in a 304 stainless steel can, then sealed after vacuum degassing to 5 × 10^−3^ Pa at 500 °C for 18 h, to further remove moisture and the remaining gas in the mixed powders. Thirdly, the sealed can was hot isostatic pressed (HIP) at 1120 °C and 140 MPa for 3 h. Fourthly, the HIPped composite was followed by isothermal forging at 1100 °C with a forging ratio of 1.7. Finally, before microstructure analysis and mechanical properties testing, all composites were subjected to a solution heat treatment at 1140 °C for 2 h and aging treatment at 870 °C for 1 h.

The morphologies and microstructures were examined using an optical microscope (OM) (DM2000, Leica Ltd., Weztlar, Germany) and field-emission SEM (Hitachi S-4800, Hitachi Ltd., Tokyo, Japan) equipped with an energy-dispersive X-ray spectrometer (EDS). The GNSs structures was observed via TEM (FEI Tecnai G^2^ F30, FEI Ltd., Hillsboro, USA) with an accelerating voltage of 200 kv. Also, the interfaces between FGH95 and GNSs were carried on through high-resolution transmission electron microscopy (HRTEM) with a FEI Tecnai G^2^ F30. Tensile properties at room temperature and 650 °C were tested to investigate the mechanical properties of samples on a testing machine (Instron 5887, Instron Co., Boston, USA). For every composite, three tensile samples were tested to reduce experimental errors. Before the tensile testing, all samples were held at the test temperature for 15 min.

## 3. Results and Discussion

### 3.1. Microstructures of Graphene Nanosheets (GNSs)/FGH95 Composite

**Figure 2:**
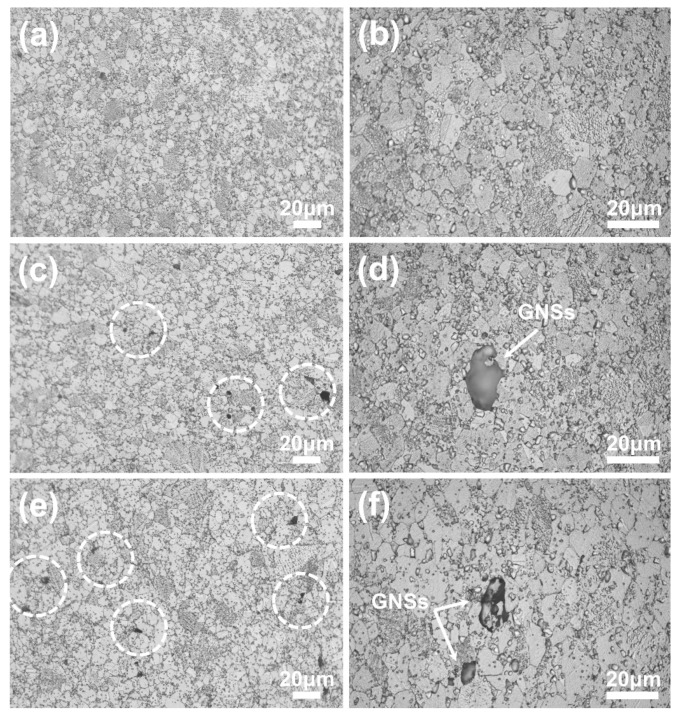
Optical microscopy (OM) images of FGH95 (**a**,**b**); 0.01 wt.% GNS-reinforced FGH95 (**c**,**d**) and 0.03 wt.% GNS-reinforced FGH95 (**e**,**f**).

OM micrographs of FGH95 and GNSs/FGH95 composites are illustrated in Figure 2. It can be found that the FGH95 and GNS-reinforced FGH95 composite are both composed of equiaxed crystals. It also can be seen that with the increasing content of GNSs, GNSs appears in the composite material and distribute along the grain boundary or across the grain boundary, showing a semi-translucent sheet-like morphology. By comparing Figure 2c,e, the distribution of GNSs in the matrix increases (as shown by the white dotted box in the figures) with the increasing of the GNSs content. The grain sizes of FGH95, 0.01 wt.% GNSs/FGH95 and 0.03 wt.% GNSs/FGH95 are very similar, which is about 7 μm. This means that the addition of GNSs has no effect on the superalloy structure, and shows a good compatibility with the superalloy matrix.

**Figure 3:**
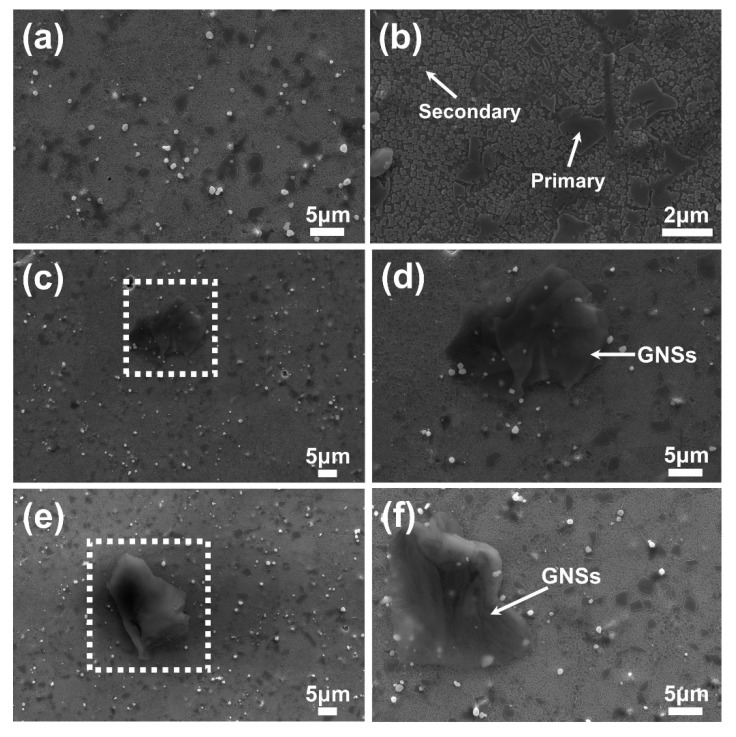
SEM images of FGH95 (**a**,**b**); 0.01 wt.% GNS-reinforced FGH95 (**c**,**d**) and 0.03 wt.% GNS-reinforced FGH95 (**e**,**f**) through electro-polished at 15 V, 1 A for 15 s and electro-etched at 2.5 V, 2 A for 1 s at room temperature.

Figure 3 illustrate the microstructure images of FGH95, 0.01 wt.% GNSs/FGH95, and 0.03 wt.% GNSs/FGH95 samples observed by SEM. It can be seen that the microstructure of the FGH95 and GNSs/FGH95 composite consisted of a primary γ’ and secondary γʹ phase (as shown by the white arrow in Figure 3b), which is the typical microstructure of nickel based superalloy FGH95 after heat treating. As shown in Figure 3c–f, no pores and holes have been detected in the GNSs/FGH95. GNSs appears to be lamellar- and film-like shape (as indicated by the white arrow in Figure 3d,f), which distribute in and across the grain boundary. After a series of thermal processes, GNSs still retains the original morphological features. It can be ascertained that the preparation process consist of blending, degassing, HIP and HIF is an effective way to fabricate GNSs/FGH95 composite. Homogeneously distributed GNSs [37] and uniformly distributed grain sizes [38] play an important role in uniform mechanical properties of graphene-reinforced composites.

**Figure 4:**
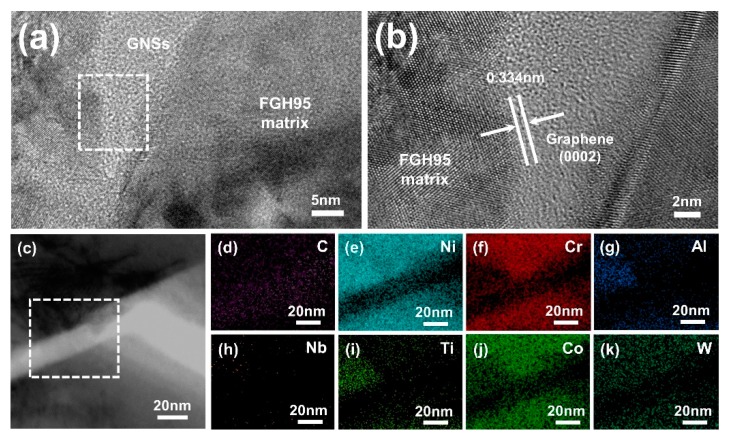
TEM images of 0.01 wt.% GNSs reinforced FGH95 (**a**) and high-resolution TEM (HRTEM) image (**b**) of the white dashed box in Figure 4a; energy-dispersive X-ray spectrometer (EDS) image (**c**) and selected corresponding EDS mappings of C (**d**), Ni (**e**), Cr (**f**), Al (**g**), Nb (**h**), Ti (**i**), Co (**j**) and W (**k**) elements for a typical GNSs/FGH95 interface.

The bright-field TEM and EDS mapping images of representative interface of GNSs reinforced FGH95 composite have been shown in Figure 4. The TEM images (as shown in Figure 4a,b) exhibit the distribution and morphology of GNSs in the FGH95 matrix. It is found that GNSs located around the boundary of grains and distributed evenly throughout the matrix. GNSs still maintain their original lamellar morphology in the composite matrix and the lattice spacing of 0.34 nm corresponds to the (0002) crystal plane of grapheme [39]. Furthermore, the HRTEM image in order to characterize the interface microstructure has shown that the GNSs and FGH95 metal matrix is well bonded with no interfacial gap such as micropores and microcracks between reinforcements and metal matrix, which is beneficial for increasing the performance of the composite. Moreover, the GNSs show a certain curly morphology, which contributes to the mechanical combination between FGH95 matrix and GNSs. Figure 4c illustrates the TEM-EDS mapping of the GNSs/FGH95 interface. Figure 4d,e–k correspond to the carbon element in the GNSs and the metal elements in the superalloy matrix, respectively. By the results of the element mapping of carbon and metals, it is verified that the GNSs is sandwiched between the FGH95 matrix. Carbon element is enriched in the middle of the EDS mapping image, and metal elements tend to diffuse into the GNSs from both sides. It is indicated that there is a diffusive interface between the reinforcement phase and the superalloy matrix, which is a strong binding interface. This combination structure can effectively transfer the load from the FGH95 matrix to GNSs, resulting in the increase of mechanical performance. Also, the hard GNSs could hinder the dislocation movement [40], leading to increased dislocation density [41] and thus enhanced mechanical properties. The microstructure observation confirmed the fine interface combination between FGH95 matrix and GNSs, which proved the effectiveness of the treatment process.

### 3.2. Mechanical Properties of GNSs/FGH95 Composite

**Figure 5:**
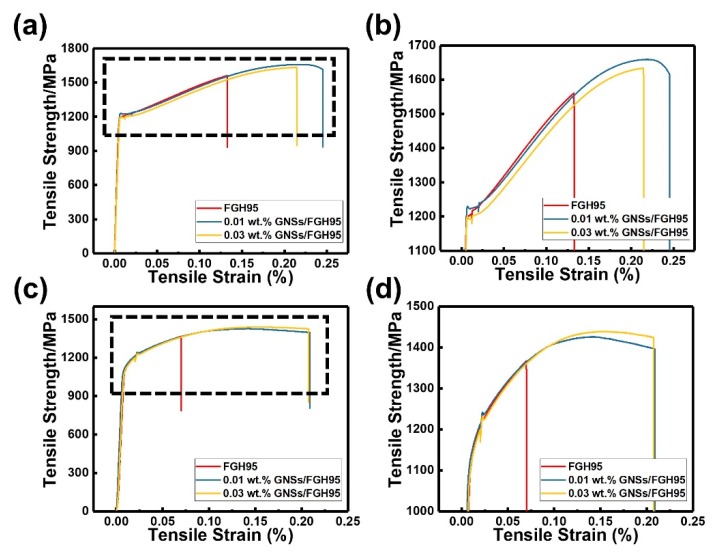
Engineering tensile stress-strain curves of FGH95, 0.01 wt.% GNSs/FGH95 and 0.03 wt.% FGH95/GNSs at room temperature (**a**,**b**) and 650 °C (**c**,**d**).

Figure 5 presents the typical mechanical engineering stress-strain curves at room temperature and 650 °C of FGH95 matrix and GNSs/FGH95 composites with 0.01 wt.% and 0.03 wt.% GNSs contents. The detailed data are listed in Table 1.

It can be seen that GNSs is an effective reinforcement in the superalloy FGH95 matrix. It has been found that the UTS, YS, elongation and reduction of area (R/A) are increased with the addition of GNSs. At room temperature, with the addition of 0.01 wt.% GNSs, the tensile strength of GNSs/FGH95 metal matrix increases from 1561 MPa to 1660 MPa with an elevation of elongation from 10.8% to 22.2%. However, when the content of GNSs reaches to 0.03 wt.%, the strength and plasticity are increased compared with FGH95 without GNSs while reduced compared with the composite material with a GNSs content of 0.01 wt.%. The increase in the strength of the FGH95 matrix material is closely related to the addition of the reinforcements of GNSs, and the main strength mechanism may be associated with the microstructure of GNSs, interface structure of GNSs and FGH95 and dispersion enhancement of GNSs. Also, at 650 °C, with adding GNSs at 0.01 wt.%, the tensile strength of GNSs/FGH95 metal matrix increases from 1367 MPa to 1426 MPa with an elevation of elongation from 8.1% to 19.4%. By contrast with room temperature, the plasticity still shows an upward trend, as the GNSs content increases to 0.03 wt.% at 650 °C. An increase of around 162% in elongation and 101% in R/A is shown. It can be observed that the tensile strength of FGH95 and GNSs/FGH95 composites decreases with the temperature increasing from room temperature to 650 °C. This phenomenon can be explained by the diffusion-controlled creep phenomenon [42] and thermal activation [43] of the GNSs/FGH95 composite, which indicates that high temperature will reduce the work-hardening effect and make plastic deformation easier. Compared with the FGH95 alloy matrix, GNSs in GNSs/FGH95 composites has strengthened the FGH95 superalloy at room temperature and 650 °C. This may be due to the uniform dispersibility [44] and the bonding ability with the metal matrix [45] of GNSs at room temperature and high temperature. These effects make GNSs act as a certain load transfer role in the composites. In addition, GNSs plays an role in pinning [46] object at the grain boundaries of FGH95 alloy and preventing grain boundary slippage and dislocation movement.

### 3.3. Fracture Behavior and Failure Mechanisms at Different Temperature

**Figure 6:**
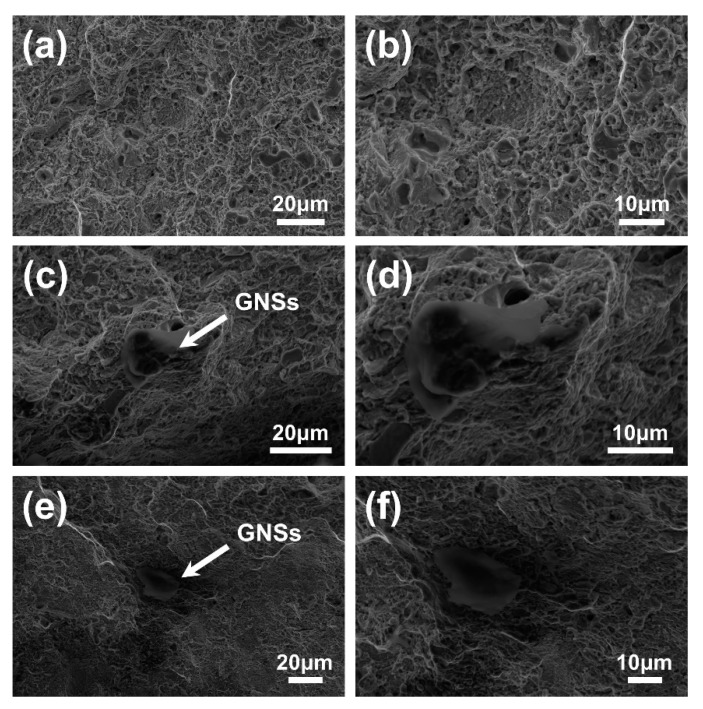
The SEM images of fractured surfaces of the FGH95 (**a**,**b**) and GNSs/FGH95 ((**c**,**d**) for 0.01 wt.% GNSs content and (**e**,**f**) for 0.03 wt.% GNSs content) composite at room temperature.

**Figure 7:**
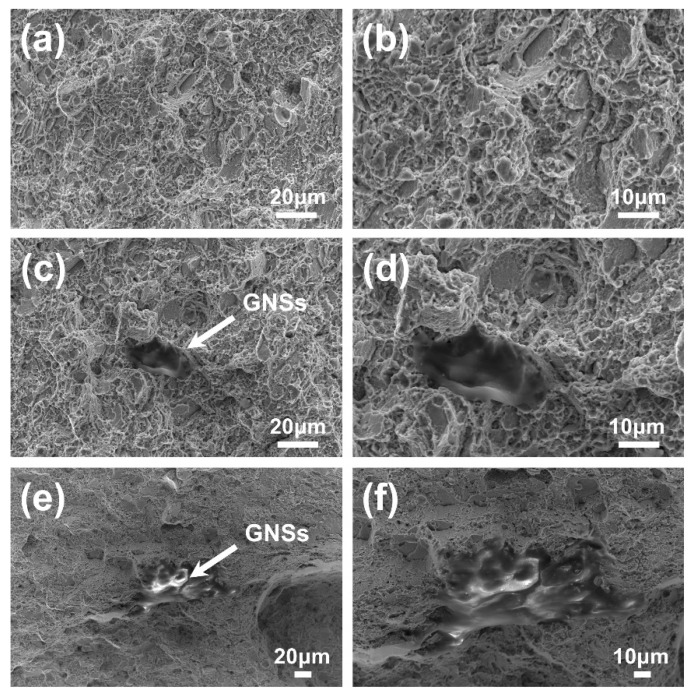
The SEM images of fractured surfaces of the FGH95 (**a**,**b**) and GNSs/FGH95 ((**c**,**d**) for 0.01 wt.% GNSs content and (**e**,**f**) for 0.03 wt.% GNSs content) composite at 650 °C.

The fracture surfaces of GNSs/FGH95 and FGH95 at room temperature and 650 °C have been shown in Figure 6 and Figure 7, respectively. As illustrated in Figure 6, the representative fracture surface of GNSs/FGH95 at room temperature shows dimpled and tear ridged morphology with visible GNSs existing in it, which indicates an effective metallurgical bonding between FGH95 matrix and GNSs. Some pulled-out GNSs can be found along the edges of tear ridges, in GNSs/FGH95 composite matrix. It can be seen from the SEM images that the GNSs located at the fracture surface have not denatured and shows initially two-dimensional wrinkled film-like structure. Owing to the supreme strength of graphene, GNSs is more likely to be pulled out from the metal matrix, rather than be destroyed on its own. As illustrated in Figure 7, the roughness of the fracture surface decreases as the temperature rises. Pulled-out GNSs from composite matrix with different morphologies can be seen on the fracture surfaces of all samples (Figure 6c,e and Figure 7c,e), after tensile failure occurred at both room temperature and 650 °C. When the tensile tests were performed at room temperature, the pulled-out GNSs show a rough morphology which turns into a smooth one at 650 °C, and the pulled-out GNSs at both temperatures have a certain curly shape.

**Figure 8:**
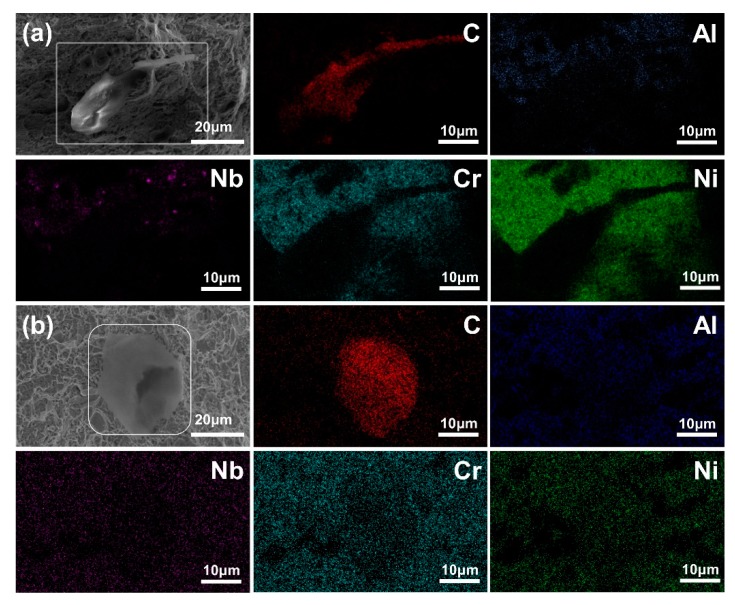
The SEM and selected SEM-EDS mapping images of fractured surfaces of the 0.01 wt.% GNSs/FGH95 (**a**) at room temperature and 0.01 wt.% GNSs/FGH95 (**b**) composite at 650 °C with carbon elements in GNSs and main FGH95 alloy elements e.g., Al, Nb, Cr and Ni.

To identify the difference of failure modes of GNSs/FGH95 between room temperature and high temperature, the fracture morphologies of the composites at both temperatures were observed by SEM and EDS, as illustrated in Figure 8. At room temperature, the pulled-out GNSs can be described as follows: (1) the pulled-out GNSs maintains the original rough morphology; (2) it has a complete edge structure; (3) it has a clean surface and the alloying elements contained in FGH95 cannot be detected (as shown in Figure 8a). In contrast, when tested at 650 °C, GNSs at the fracture surface show different morphology and composition. The pulled-out GNSs have a relatively smooth morphology. Through EDS testing, it can be found that the surface of the pulled-out GNSs is covered by the alloying elements in FGH95, as illustrated in Figure 8b with the EDS elements mapping. Therefore, the FGH95 matrix around GNSs has been destroyed at high temperature, which means that the shear strength of the GNSs/FGH95 interface is higher than that of the FGH95 matrix at 650 °C.

**Figure 9:**
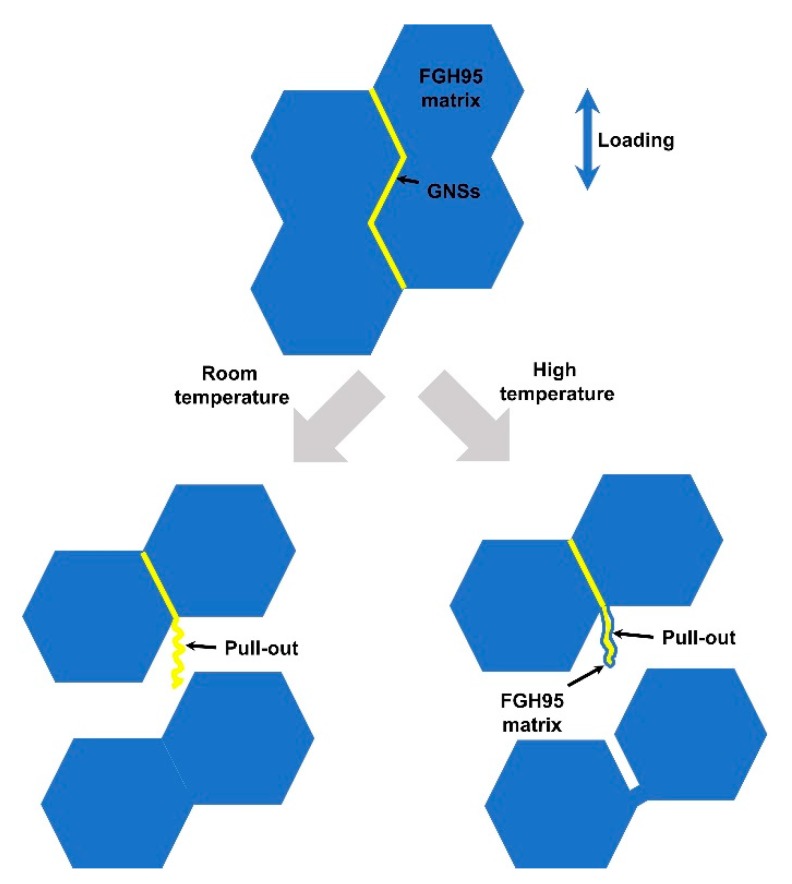
Schematic illustration of GNSs’s failure mode in the GNSs/FGH95 composites at room temperature and high temperature (650 °C).

According to the above results, the failure modes of GNSs/FGH95 at room temperature and high temperature can be summarized as shown in Figure 9. At room temperature, the interfacial debonding can be regarded as the main failure mode. No alloying elements in FGH95 have been detected on the GNSs surface. Due to the instantaneous release of stress, the GNSs rebounds instantly to show a certain curled shape after being pulled out. In contrast, at 650 °C, the failure mode of GNSs/FGH95 composites is caused by the fracture of the FGH95 matrix around the interface. The pulled-out GNSs has not been broken and covers the alloying elements in FGH95. The pulled out GNSs present a similar curling structure, because of the fact that the fracture is a transient process whether stretched at room temperature or high temperature. This also explains that as the content of GNSs increases, the mechanical properties at room temperature and high temperature show different enhancement trends. At room temperature, the less interaction between GNSs and FGH95 matrix results in the plasticity decreasing when the content increases. At high temperature, the interaction between GNSs and FGH95 matrix is enhanced and the increase of GNSs content provides more interaction sites. Therefore, at high temperature, the plasticity also increases with the increase of GNSs content.

Considering that adding only a small amount of GNSs (<0.03 wt.%) has significantly improved the mechanical performance of the composites, GNSs has higher efficiency in improving mechanical performance than other reinforcements [6,29]. It is demonstrated that GNS, as an ideal reinforcement, has enormous potential applications in nickel-based superalloy FGH95.

## 4. Conclusions

In this study, GNS-reinforced nickel-based superalloy FGH95 composite was successfully prepared by wet chemical method combined with hot isostatic pressing. The mechanical properties and failure mechanisms of GNSs/FGH95 were investigated at room temperature and high temperature. In both cases, GNSs played an important role in improving the mechanical performance of GNSs/FGH95. The softening of the matrix caused by temperature rise was the main reason for the high temperature strength to be lower than the room temperature strength. GNSs was observed to be pulled out from composite matrix at both room temperature and high temperature. However, the fracture mechanism was not the same. Specifically, the interface debonding was considered to be the dominant factor at room temperature, while the matrix rupture was the main reason at high temperature. FGH95 nickel-based superalloys have been widely used in aeroengine structural materials. The experimental results are of great significance for the design of GNS-reinforced nickel-based composites with high thermal structural stability and wide application at high temperatures.

## Figures and Tables

**Table 1:** The specific mechanical properties of FGH95, 0.01 wt.% GNSs/FGH95 and 0.03 wt.% FGH95/GNSs at room temperature and 650 °C.

Sample	Temperature (°C)	UTS (MPa)	YS (MPa)	Elongation (%)	R/A (%)
FGH95	23	1561 ± 15	1167 ± 13	10.8 ± 1.3	12.0 ± 1.2
0.01 wt.% GNSs/FGH95		1660 ± 17	1229 ± 11	22.2 ± 1.5	29.9 ± 1.6
0.03 wt.% GNSs/FGH95		1634 ± 14	1198 ± 16	19.2 ± 1.1	21.5 ± 1.0
FGH95	650	1367 ± 16	1068 ± 11	8.1 ± 1.5	10.9 ± 1.3
0.01 wt.% GNSs/FGH95		1426 ± 14	1110 ± 13	19.4 ± 0.9	20.1 ± 1.1
0.03 wt.% GNSs/FGH95		1439 ± 11	1106 ± 13	21.3 ± 1.1	21.9 ± 1.2
